# Impacts of Sediments on Coral Energetics: Partitioning the Effects of Turbidity and Settling Particles

**DOI:** 10.1371/journal.pone.0107195

**Published:** 2014-09-08

**Authors:** Reef K. Junjie, Nicola K. Browne, Paul L. A. Erftemeijer, Peter A. Todd

**Affiliations:** 1 Experimental Marine and Ecology Laboratory, Department of Biological Sciences, National University of Singapore, Singapore, Singapore; 2 Department of Environment and Agriculture, Curtin University, Perth, Australia; 3 Sinclair Knight Merz (SKM), Perth, Australia; 4 The UWA Oceans Institute, University of Western Australia, Crawley, Australia; 5 Tropical Marine Science Institute, National University of Singapore, Singapore, Singapore; New England Aquarium, United States of America

## Abstract

Sediment loads have long been known to be deleterious to corals, but the effects of turbidity and settling particles have not previously been partitioned. This study provides a novel approach using inert silicon carbide powder to partition and quantify the mechanical effects of sediment settling versus reduced light under a chronically high sedimentary regime on two turbid water corals commonly found in Singapore (*Galaxea fascicularis* and *Goniopora somaliensis*). Coral fragments were evenly distributed among three treatments: an open control (30% ambient PAR), a shaded control (15% ambient PAR) and sediment treatment (15% ambient PAR; 26.4 mg cm^−2^ day^−1^). The rate of photosynthesis and respiration, and the dark-adapted quantum yield were measured once a week for four weeks. By week four, the photosynthesis to respiration ratio (P/R ratio) and the photosynthetic yield (F_v_/F_m_) had fallen by 14% and 3–17% respectively in the shaded control, contrasting with corals exposed to sediments whose P/R ratio and yield had declined by 21% and 18–34% respectively. The differences in rates between the shaded control and the sediment treatment were attributed to the mechanical effects of sediment deposition. The physiological response to sediment stress differed between species with *G. fascicularis* experiencing a greater decline in the net photosynthetic yield (13%) than *G. somaliensis* (9.5%), but a smaller increase in the respiration rates (*G. fascicularis* = 9.9%, *G. somaliensis* = 14.2%). These different physiological responses were attributed, in part, to coral morphology and highlighted key physiological processes that drive species distribution along high to low turbidity and depositional gradients.

## Introduction

Singapore's diverse coral reef system hosts 255 hard coral species [1], [2] but pressure from coastal reclamation and dredging operations have resulted in the loss of at least 60% of its original coral reef area [3]. It is estimated that poor water quality associated with high sediment loads will further reduce Singapore's original reef to only 21% by 2030 [4]. Since the 1970s, sedimentation rates have risen from 3.2–5.9 mg cm^−2^ d^−1^ [5] to 15–30 mg cm^−2^ d^−1^ [6], [7], [8] with average visibility reduced from 10 m (1960s) to less than 2 m [9]. Sedimentation rates and turbidity decline with increasing distance from Singapore's mainland, resulting in spatial variations in coral species composition and reef health [10]. The least threatened and most diverse coral reefs are those furthest to the south (e.g. at Raffles light house which supports 141 hard coral species).

Inshore to offshore changes in coral cover and/or composition observed in numerous coral reef systems, including the Great Barrier Reef, are in part attributed to spatial differences in the sediment regime [11], [12], [13]. Coral species better adapted or acclimatised to cope with the stress effects of down-welling sediment are typically more abundant in reef habitats exposed to high sediment loads [14], [15], [16], [17]. Increased sediment deposition can cause coral mortality by smothering and burying [18], limit hard substrate availability and decrease larvae settlement rates, increase energy costs due to active sediment removal [19] and reduce energy available for coral calcification [20], [21] and reproduction, and promote tissue infection [22], [23], [24]. High sediment loads usually result in greater turbidity, limiting light availability and reducing photosynthetic yield by symbiotic zooxanthellae. This results in a decrease in net productivity and lower carbon gain. Anthony and Hoegh-Guldberg [25] showed that it is possible for zooxanthellae to photo-acclimatize by increasing the number and size of chloroplasts. This response varies among species with some corals being better able to photo-acclimate, and thus capable of growing at deeper sites or on turbid reefs typically found close to shore.

Many corals on turbid reefs have developed morphological as well as physiological mechanisms that have enabled them to survive the negative effects of high sediment loads. For example, *Turbinaria* develops a funnel shape which directs sediments to the colony base reducing the area affected by sediment [26] whereas other corals have developed ways to rapidly remove sediment either through polyp projection (e.g. *Goniopora*), ciliary action (e.g. *Galaxea*) or mucus production [27], [28]. However, the removal of sediments comes at an energetic cost to the affected corals [29]. Under chronically high sedimentation and turbidity regimes, or during acute sediment stress events such as dredging operations, corals experience limited light and energy capture coupled with increased energy expenditure, which may exceed coral tolerances and lead to tissue mortality [30]. The point when coral tolerances are exceeded, and the physiological changes that occur within the coral due to both the separate and combined effects of reduced light and sediment smothering, are not fully understood. This uncertainty is due to the number of associated effects of sediments that go beyond shading the coral, most of which are negative: e.g. the sediment barrier prevents gas exchange and waste removal [19], and nutrients and organics in sediment promote rapid increases in bacterial populations in coral mucus [31]. However, sediments may also provide an additional food source through heterotrophic feeding, offsetting the carbon deficit [32]. Coral responses to sediments differ significantly both among and within coral species [27], [30], further complicating the analysis.

The balance between sedimentation and sediment resuspension is considered to be a key driver of coral community composition and distribution on turbid coral reefs [33], [34], [35], [36], [37], [38]. Numerous studies have attempted to understand the biological response of corals to sediment stress by measuring growth rates, photosynthetic yields, the ratio of photosynthesis to respiration (P/R ratio), and tissue mortality. For example, a study by Crabbe and Smith [38] on turbid reefs in south-east Sulawesi, Indonesia, found lower growth rates of both branching and non-branching corals with increased sedimentation, while Weber et al. [39] measured reduced photosynthetic yields within 48 hrs of sediments settling on corals. In the latter study, sediments with higher organic content (>0.5%) caused the largest reductions in the photosynthetic yield (>30% within 48 hrs). Exposure to sediments will also decrease the P/R ratio [30], [40], [41], however, there are disagreements within the literature as to whether the reduction in the P/R ratio is largely driven by light-limited photosynthesis or increased respiration rates associated with removing settling sediments. If the former is correct, then photo-efficiency together with heterotrophic capacity would drive the partitioning of species between reef habitats characterised by high sediment resuspension rates versus habitats dominated by sediment deposition [42]. However, Anthony and Connolly [43] suggest that energy costs associated with handling sediment stress, e.g. sediment clearing [29] and increased respiration, drive coral sediment tolerances and hence species distribution between resuspension and depositional environments. What remains unclear is how coral physiology is influenced separately by turbidity and limited light penetration versus the physical effects of sediments settling on corals and subsequent sediment clearing.

The present study used a laboratory-based approach to determine the physiological effects of sediment deposition, independent of light, on two coral species (*G. fascicularis, G.somaliensis*) commonly found on turbid water reefs in the Indo-Pacific. The experimental setup was designed to deliver sediments at a constant chronic rate over several weeks that resulted in a sedimentation rate typically measured on turbid reefs. Inert silicon carbide powder (10 µm to 300 µm), rather than natural reef material, was used in the study to assess the mechanical effects of down-welling particles on corals without confounding factors such as nutrients, heavy metals and microbes found in natural sediments [44]. The effects of sedimentation on coral physiology were examined and compared with two controls: open and shaded. The light levels in the shaded control were carefully aligned with the light levels in the sediment exposed treatment, thereby allowing the comparative assessment of reduced light versus reduced light and sedimentation. The physiological response of corals to sediment deposition and light reduction were measured using P/R ratios and photosynthetic yields. The two primary objectives of the study were to: 1) quantify the physiological effects of chronic sedimentation for two common Indo-Pacific corals, and 2) assess the difference in coral physiological response between the mechanical effects of sediment deposition and light reduction.

## Materials and Methods

### Study species and sampling design

*Galaxea fascicularis* and *Goniopora somaliensis* are commonly found in sheltered reef environments throughout the Indo-Pacific [45]. *G. fascicularis* grows into domed colonies with cylindrical corallites (<10 mm diameter) linked together by horizontal plates (coenosteum) [45]. In contrast, *G. somaliensis* forms sub-massive colonies with shallow calices and small corallites (3 to 5 mm diameter) with a smooth surface [45]. Both species are hermatypic, meeting the majority of their energy demands through photosynthesis by the zooxanthellae living within their tissues. Research permit (No. NP/RP12-007), that includes permission to collect corals, was granted by the National Parks Board (NParks), a statutory government board in charge of terrestrial and marine parks in Singapore, under the project entitled ‘Impacts of ship-wake induced sediment resuspension on corals reefs and sea grass in Singapore.”

In December 2011, fragments (surface area = 95±2 cm^2^, determined using the ‘aluminium foil’ method [46]) were chiselled off 12 colonies of *G. fascicularis* and 12 colonies of *G. somaliensis* found at ∼3 m depth at Lowest Astronomical Tide (LAT) at the north-western fringing reef of Pulau Hantu (103.44°E, 1.13°N): an island located 8 km southwest of Singapore's main island. In the mid-1970s much of the reef flat surrounding Pulau Hantu was destroyed following a reclamation project which increased the island's land area from 0.024 to 0.12 km^2^ [2]. Today, approximately 2.21 km^2^ of intertidal reef flat remains [47]. Sedimentation rates on the reef slope often exceed 10 mg cm^−2^ d^−1^ [6], [48], [49], levels considered to be detrimental to the more sensitive species of coral [40]. At 3 m depth (LAT), turbidity typically ranges between 5 and 20 mg l^−1^ and light levels vary from 50 to 200 mol photons m^−2^ s^−1^ [48]. All fragments were maintained in a holding tank supplied with continuous flow of sand-filtered seawater at the Tropical Marine Science Institute research facility on St John's Island, south of mainland Singapore (193.85°E, 1.22°N). Corals were left to acclimatize for three weeks before the experiment commenced in January 2012.

### Experimental setup and design

**Figure 1:**
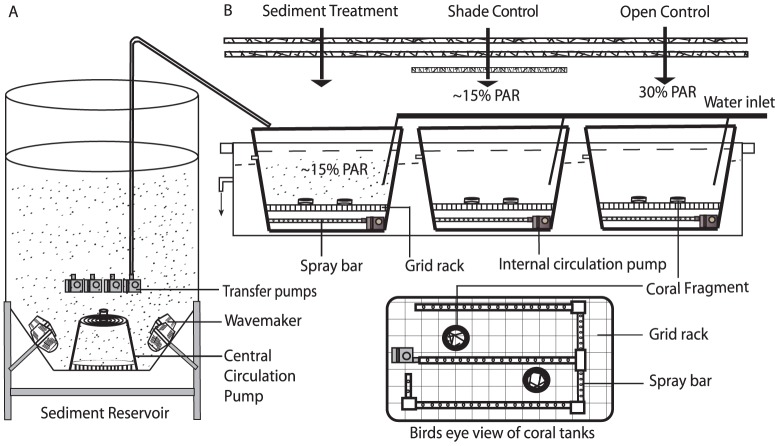
Experimental setup. (A) Sediment reservoir, (B) Coral treatment tanks. Four tanks for each treatment (sediment, shade, open) were placed in a water bath positioned under a shade cloth which reduced ambient light to 30%. Additional shading was placed over the shaded to control which further reduced ambient light levels to 15%, comparable light levels to that of the sediment treatment tanks.

The experimental setup, adapted from Anthony [50], comprised of 12 tanks (42 L: 45×30×31 cm) with three different treatments: open control, shaded control, and sediment-stressed (Fig. 1). Each tank received a constant supply of fresh sand-filtered seawater through transfer pumps from the sediment reservoir at a rate of ∼350 cm^3^ min^−1^. An internal convection current at the bottom of each sediment treatment tank prevented immediate settlement of sediment through the use of a spray bar attached to an internal circulation pump (Boyu 1200 L h^−1^, China). The tanks were placed in a fiberglass water bath (1.6×1.5×0.3 m) with continuous water flow to ensure uniform water temperature in all the tanks (∼28°C; temperatures typically experienced in Singapore waters). Following acclimatization, one fragment from each species was randomly selected and placed in the treatment tanks on a plastic grid to prevent accumulation of sediments at the base of each coral fragment. Fragments were rotated between the four corners within each tank once per week for four weeks.

### Sediment preparation and delivery

**Figure 2:**
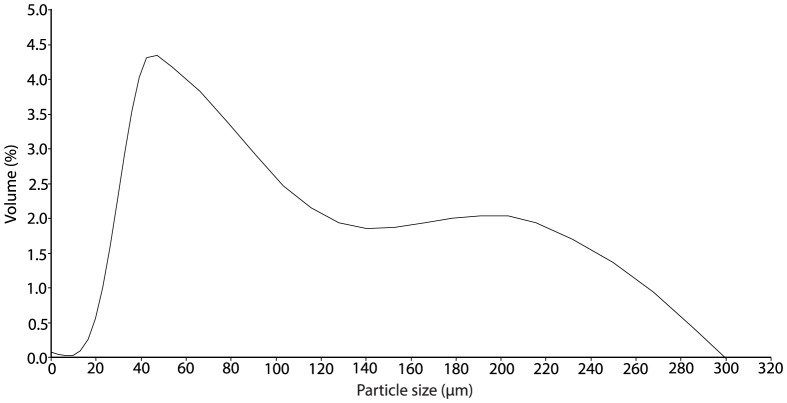
The sediment particle profile of the silicon carbide powder.

The grain size distribution of the natural sedimentation profile at Pulau Hantu was analysed by Lui et al. [44] through laser diffraction particle size analyses (Malvern Mastersizer Particle Size Analyser, UK) and matched using a combination of commercially available particle sizes of silicon carbide powder (Kemet Far East Pte Ltd). Silicon carbide, also known as carborundum, is chemically inert and has been previously used in coral sediment rejection studies [19]. The final silicon carbide mix contained particle sizes ranging from 10 µm to 300 µm, and had a median particle size of 60 µm (Fig. 2).

A 250 L tapered bottom cylindrical fibre-glass drum was fabricated and used as the sediment suspension reservoir, providing material to the four sediment-stressed tanks. Silicon carbide in the drum was kept in suspension and homogenized by a central circulation pump and two aquarium wave-makers (Sun Sun 6000 L h^−1^, China; Fig. 1). The sediment-suspension (75 ml) was supplied to the sediment-stress treatment tanks, via four transfer pumps (BOYU 1200 L h^−1^, China), in discrete 3 s pulses every 10 minutes, which resulted in a relatively constant sediment “rain” in the tanks. The sedimentation rate was measured by placing three petri dishes (75 mm diameter) in each sediment-stress tank for 24 h, and the mean sedimentation rates for the four individual tanks were: 26.6±0.28, 26.3±0.38, 25.8±0.55 and 27.0±0.18 mg cm^−2^ day^−1^.

### Light measurements and attenuation

The experiment design was setup outdoors underneath shading nets that reduced solar light penetration to ∼30% of ambient light. In January 2012, the daily surface irradiance in the open control ranged from 600 to 1800 mol photons m^−2^ s^−1^ (measured in the tanks at the level of the coral fragments) between 10 am and 2 pm. Prior to the start of the experiment, light measurements using LI-1400 Data logger (LI-COR, United States) indicated that light levels within the sediment stress tanks further reduced light penetration to ∼50% of surface irradiation. Hence, additional shade netting was added to create the shade control tanks so that they also received ∼15% ambient PAR (Fig. 1).

We acknowledge that when sediments settle on corals at a rate that far exceeds their removal rate (during rapid sediment loading events e.g. dredging) the sediment layer creates an additional barrier to light [51], further limiting photosynthesis. However, in this chronic sedimentary regime, the level of sediment deposition (∼26 mg cm^−2^ day^−1^) was well below previous measured rates that may lead to light attenuation at the surface (200 mg cm^−2^ leads to a 75% light reduction at the surface [29]) and the two coral species were able to remove sediments from their surfaces relatively quickly. Hence, we are confident that light levels at the coral surface in both the shaded control and sediment treatment tanks were comparable.

### Physiological assessment

Two physiological parameters—P/R ratio and photosynthetic yield—were used as quantitative indicators of coral stress. The maximum photosynthetic yield (Fv/Fm) for all 24 coral fragments was measured weekly between 1 and 3 pm using a Diving-PAM, Walz Germany [52]. The coral fragments were dark-adapted for 10 min before fluorescence yield measurements were taken [53], [54]. The optical-fiber probe was kept at a constant distance of 5 mm from the surface of the coral and the average of five measurements for each coral fragment was calculated. F_o_ was measured by applying a pulsed measuring beam of <1 µmol photon m^−2^ s^−1^ and the emission F_m_ was measured following the application of a saturating pulse of actinic light (>1000 µmol photon m^−2^ s^−1^). The P/R ratio was measured using RESP-EDU software and Loligo Systems respiratory system (Denmark) between 10 am and 12 pm. A circular respirometry chamber (1.5 L) with a circulatory pump and an integrated galvanic cell oxygen probe (Loligo Systems, Denmark), was fabricated to accommodate the size of the fragments. Oxygen consumption/production (mg O_2_ l^−1^) within the chamber (after subtracting the volume of coral) was measured every 1 minute for 5 minutes, followed by a 2 min flush period. The net photosynthetic rates were measured after 2 hrs of solar light irradiance (295±10 mol photons m^−2^ s^−1^; ambient light under shading) whereas respiratory rates were measured after 10 min incubation in the dark. Oxygen production and respiration rates were normalised to the surface area of live tissue for each coral fragment (µmol cm^−2^ hr^−1^), and the P/R ratio was calculated by dividing the gross photosynthetic rate (the net photosynthetic rate added to the respiration rate) by the respiration rate.

### Statistical analyses

All statistical analyses were performed using IBM SPSS Statistics Standard v. 19 (2011), comparing the percentage change in photosynthesis, respiration and yield between treatments over the four weeks. Percentage change, as opposed to absolute values, was used as coral fragments in week 1 had marginally variable baseline photosynthesis, respiration and yield values. Hence, absolute values averaged over replicates could obscure trends. Oxygen production and respiration (µmol cm^−2^ hr^−1^) and yield measurements (Fv/Fm) were converted to a percentage change by comparing the rate in weeks 2, 3 and 4, to rates and yields collected in week 1. Negative values of percentage change indicated a decline in the rate and yield. Data were checked for normality and homogeneity of variance using the Shapiro-Wilk test and Levene's test respectively. One-way repeated measures ANOVAs (α = 0.05) were performed (n = 4), with adjustments made for multiple comparisons using Bonferroni corrections, to assess if and when there was a significant change in rates and yields over the four weeks for each of the controls (open and shaded) and the sediment-stressed treatment. Mauchy's test of sphericity was carried out, and where the assumption was violated, data was adjusted using the Greenhouse Geisser adjustment. Power calculations were also performed in SPSS, by selecting the relevant option during the repeated measures analysis process, to verify differences in samples were representative (>0.8) of differences in the population.

## Results

### Photosynthesis and respiration

**Figure 3:**
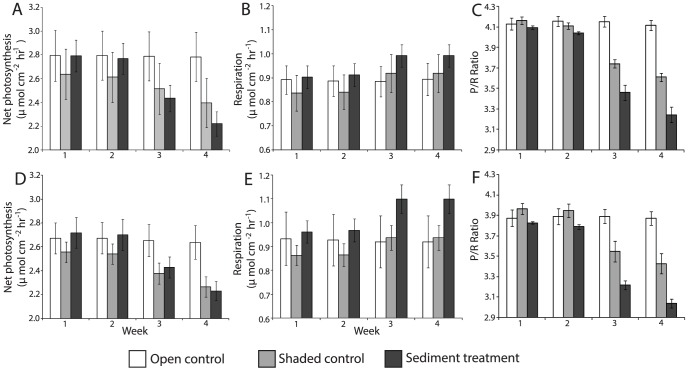
Net photosynthetic rates, the respiration rates and the P/R values for *G.fascicularis* (A, B, C) and *G.somaliensis* (D, E, F) over four weeks in the open control (white), shaded control (grey) and sediment treatment (black). The error bars represent standard errors (SE), and n = 4.

The net photosynthetic rate for *G. fascicularis* and *G. somaliensis* in the shaded control and sediment treatments fell from >2.5 µmol cm^−2^ hr^−1^, measured at the start of the experiment, to approximately 2.4 µmol cm^−2^ hr^−1^ by week 3 and to 2.2–2.4 µmol cm^−2^ hr^−1^ by week 4 (Fig. 3; Table 1). The decline in photosynthesis was significant for both coral species (p<0.022; Table 2), and post hoc analysis revealed that the most significant decline occurred between weeks 3 and 4 (p<0.001; Table 3).

**Table 1:** Absolute means and percentage change values of net photosynthesis, gross photosynthesis, respiration, the P/R ratio and the yield during the course of the experiment for *G. fascicularis* and *G. somaliensis*.

Absolute values		Week 1				Week 2			
Coral	Treatment	Net photosynthesis (µmol/cm^2^/hr)	Respiration (µmol/cm^2^/hr)	Ratio	Yield	Net photosynthesis (µmol/cm^2^/hr)	Respiration (µmol/cm^2^/hr)	Ratio	Yield
*Galaxea*	Open control	2.79	0.89	4.13	0.52	2.80	0.89	4.15	0.50
	Shaded control	2.64	0.84	4.16	0.55	2.61	0.84	4.11	0.50
	Sediment	2.79	0.90	4.09	0.51	2.77	0.91	4.04	0.48
*Goniopora*	Open control	2.67	0.93	3.87	0.62	2.67	0.93	3.89	0.69
	Shaded control	2.56	0.86	3.97	0.60	2.54	0.86	3.95	0.57
	Sediment	2.72	0.96	3.83	0.60	2.70	0.97	3.79	0.54

		Week 3				Week 4			
*Galaxea*	Open control	2.79	0.88	4.15	0.50	2.78	0.89	4.11	0.52
	Shaded control	2.51	0.92	3.74	0.47	2.40	0.92	3.61	0.46
	Sediment	2.43	0.99	3.46	0.41	2.22	0.99	3.24	0.41
*Goniopora*	Open control	2.65	0.92	3.89	0.66	2.64	0.92	3.87	0.67
	Shaded control	2.38	0.94	3.55	0.54	2.26	0.94	3.43	0.58
	Sediment	2.43	1.10	3.22	0.51	2.23	1.10	3.04	0.40

Percentage change		Week 1				Week 2			
*Galaxea*	Open control	0.00	0.00	0.00	0.00	0.10	−0.57	0.64	−2.61
	Shaded control	0.00	0.00	0.00	0.00	−0.95	0.46	−1.15	−10.41
	Sediment	0.00	0.00	0.00	0.00	−0.76	1.07	−1.37	−5.79
*Goniopora*	Open control	0.00	0.00	0.00	0.00	0.08	−0.60	0.47	10.53
	Shaded control	0.00	0.00	0.00	0.00	−0.63	0.10	−0.45	−4.71
	Sediment	0.00	0.00	0.00	0.00	−0.64	0.68	−0.97	−11.23

		Week 3				Week 4			
*Galaxea*	Open control	−0.16	−0.8	0.6	−4.1	−0.4	0.3	−0.3	1.1
	Shaded control	−4.66	9.7	−10.1	−15.6	−9.1	9.7	−13.2	−17.7
	Sediment	−12.77	9.9	−15.5	−18.7	−20.4	9.9	−20.8	−19.0
*Goniopora*	Open control	−0.67	−1.4	0.4	6.7	−1.2	−1.4	−0.0	7.1
	Shaded control	−6.99	8.5	−10.5	−10.2	−11.4	8.5	−13.5	−3.1
	Sediment	−10.64	14.2	−15.9	−16.3	−17.9	14.2	−20.6	−34.1

Results are provided for the open control, shaded control and sediment treatment every week for four weeks. A negative value under percentage change indicates a drop in the associated variable compared to week one.

**Table 2:** Summary results of one-way repeated measures ANOVA (α = 0.05, n = 4) to assess if there was a significant difference in the percentage change of photosynthesis, respiration and yield by the end of the experiment (week 4).

		Open control					Shaded control					Sediment				
Coral	Measure	df	MS	F	P value	Obs Pwr	df	MS	F	P value	Obs Pwr	df	MS	F	P value	Obs Pwr
*Galaxea*	Net Photo	2	0.25	1.12	0.860	0.168	1	136	18.85	**0.022**	**.810**	1	726.46	23.90	**0.016**	**.887**
	Error	6	0.23				3	7.23				3	31.91			
	Respiration	2	2.05	2.49	0.164	0.324	1	231.83	74.62	**0.003**	**.999**	1	215.31	26.41	**0.014**	**.912**
	Error	6	0.42				3	3.11				3	8.15			
	Yield	2	29.06	1.64	0.270	0.227	2	58.51	9.74	**0.013**	**.860**	2	228.28	31.85	**0.001**	**1.00**
	Error	6	17.68				6	6.01				6	7.16			
*Goniopora*	Net Photo	2	1.95	4.75	0.058	.557	1	233.64	23.39	**0.017**	**.881**	2	587.47	161.57	**0.001**	**1.00**
	Error	6	0.41				3	10.00				6	3.64			
	Respiration	2	0.79	2.52	0.160	.328	1	187.64	300.94	**<0.001**	**1.00**	2	480.62	62.58	**0.004**	**.998**
	Error	6	0.31				3	0.62				6	7.68			
	Yield	2	16.98	1.44	0.308	.204	1	93.49	1.25	0.345	.128	2	612.45	14.79	**0.005**	**.964**
	Error	6	11.79				3	74.52				6	41.42			

Data were analysed untransformed and numbers in bold indicate p<0.05. Mauchy's test of sphericity was violated for the shaded controls and sediment treatment corals, and hence, data was adjusted using the Greenhouse Geisser adjustment.

**Table 3:** Post-hoc analysis following one-way repeated measures ANOVA to assess when (i.e. either between weeks 2 and 3, weeks 2 and 4, and weeks 3 and 4) there was a significant change in photosynthesis, respiration and yield for the shaded control and sediment treatment.

			Shaded control			Sediment		
Coral	Measure	Time	Week 2	Week 3	Week 4	Week 2	Week 3	Week 4
*Galaxea*	Net photosynthesis	Week 2		0.320	**0.046**		0.137	**0.031**
		Week 3	0.320		**<0.001**	0.137		**<0.001**
		Week 4	**0.046**	**<0.001**		**0.031**	**<0.001**	
	Respiration	Week 2		**0.010**	**0.010**		**0.043**	**0.043**
		Week 3	0.010		1.000	**0.043**		1.000
		Week 4	0.010	1.000		**0.043**	1.000	
	Yield	Week 2		0.277	0.089		**0.045**	**0.002**
		Week 3	0.277		0.230	**0.045**		1.000
		Week 4	0.089	0.230		**0.002**	1.000	
*Goniopora*	Net photosynthesis	Week 2		0.140	**0.034**		**0.011**	**0.002**
		Week 3	0.140		**<0.001**	**0.010**		**<0.001**
		Week 4	**0.034**	**<0.001**		**0.002**	**<0.001**	
	Respiration	Week 2		**0.001**	**0.001**		**0.013**	**0.013**
		Week 3	**0.001**		1.000	**0.013**		1.000
		Week 4	**0.001**	1.000		**0.013**	1.000	
	Yield	Week 2		0.284	1.000		0.680	0.078
		Week 3	0.284		1.000	0.680		0.060
		Week 4	1.000	1.000		0.078	0.060	

There was no significant difference in measured variables for corals held within the open control treatment. Numbers in bold indicate p<0.05.

In the shaded control and sediment treatment, the respiration rates increased from <0.9 in week 1 to 0.9–1.00 µmol cm^−2^ hr^−1^ measured in week 4 for *G. fascicularis*, and from <0.96 to <1.10 µmol cm^−2^ hr^−1^ for *G. somaliensis* (Fig. 3; Table 1). The respiration rate was significantly greater by week 3 in both the shaded control and sediment treatment for both coral species (p<0.043; Table 3).

**Figure 4:**
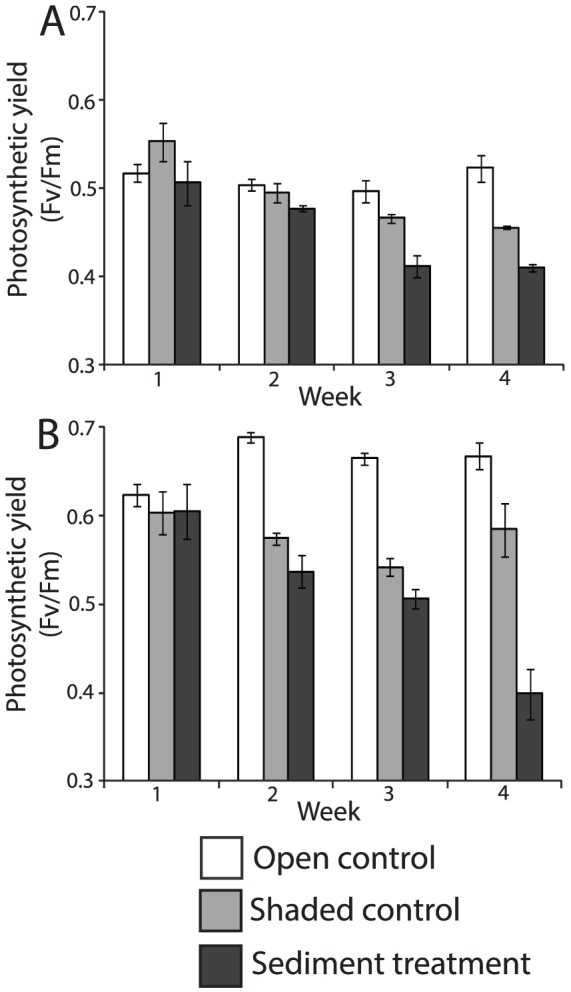
The photosynthetic yield for *G.fascicularis* (A, C) and *G.somaliensis* (B, D) over four weeks in the open control (white), shaded control (grey) and sediment treatment (black). The error bars represent standard errors (SE), and n = 4.

The P/R ratio was greater in *G. fascicularis* (range = 3.04–4.11) than in *G. somaliensis* (range = 2.94–4.05) in all treatments and over time (Fig. 4). The decline in the P/R ratio by week 4 was comparable between species with a 14% decline occurring in the shaded control and a decline of 21% in the sediment treatment (Table 1). Thus, the mechanical effects of sedimentation resulted in an additional 50% decline in coral physiological function. In contrast, the P/R ratios in the open controls were stable throughout the experiment. Despite similar declines in the P/R ratio, relative causes for the decline differed between species with lower P/R ratios largely due to reduced photosynthesis in *G. fascicularis* but the result of increased respiration in *G. somaliensis*.

### Photosynthetic yield

The photosynthetic yield (Fv/Fm) was consistently greater for *G. somaliensis* (range = 0.41 to 0.62) than for *G. fascicularis* (range = 0.41 to 0.53) within all treatments. There was no significant difference in the quantum yield in both the open and shaded control for *G. somaliensis* over the course of the experiment (Table 2). The quantum yield in the sediment treatment did, however, decline each week and was significantly lower by week 4 (0.6 to 0.41; p<0.001). In contrast, the quantum yield for *G. fascicularis* in both the shaded control (week 4; p = 0.013) and sediment treatment declined each week with significant weekly declines in the sediment treatment (Table 3). By the end of the experiment, the quantum yield had declined by 3% and 34% for *G. somaliensis*, and 17% and 19% for *G. fascicularis* in the shaded control and sediment treatment respectively, further illustrating species differences in coral physiological responses to reduced light and sediment stress.

## Discussion

This laboratory study provides the first analysis of the partitioned effects of turbidity and settling sediment on coral energetics during a chronic sedimentary event. The effects of turbidity and sediment settling were separated using a novel experimental setup whereby the effects of shading were compared with the combined effects of shading (turbidity) and settling sediment on two turbid water corals. Given that corals in the shaded control and sediment treatment experienced the same light levels, we were able to demonstrate that chronic sedimentation had a greater negative effect on corals than reduced light levels alone given that the P/R ratios and photosynthetic yield were significantly lower (p<0.04) in the sediment treatment (P/R ratio = 3.04–3.24, yield = 0.40–0.41) than both the open (P/R ratio = 3.87–4.11, yield = 0.52–0.67) and shaded controls (P/R ratio = 3.42–3.61, yield = 0.46–0.58). However, coral species responses to reduced light and the deposition of sediments on the coral surface differed, most likely due to coral morphology.

Coral respiration rates in the sediment treatment significantly increased each week, illustrating the increased energy expenditure required to remove sediments as they settle. Corals can actively remove sediments from their surfaces either through the production of mucus or by active ciliary action [41]. These processes are energetically expensive [19] and will lead to increased respiration rates, but are necessary to prevent tissue mortality under settled sediments [40], [55]. Interestingly, there was also a consistent increase in the respiration rate of those coral fragments in the shaded control (Fig. 2). Typically, under low light conditions coral respiration rates are lower than at higher light levels due to decreased energy expenditure [56], [57]. However, there have been many exceptions to this trend, with several studies showing a lack of sensitivity to light levels [42], [58]. A potential explanation for the unexpected increase in respiration rates is photo-inhibition. Hoogenboom et al. [59] demonstrated that when low light photo-acclimated corals are exposed to increased light levels, respiration increases due to photo-inhibition. In this study, corals in the shaded treatment were exposed to 15% of ambient light (typically 90–200 mol photons m^−2^ s^−1^) but respiration of dark adapted corals was measured following a two hour incubation at ∼300 mol photons m^−2^ s^−1^ for the photosynthetic measurements. The increase in light levels, even though relatively small and for a short time period, may be sufficient to stress the corals resulting in higher respiration rates. Despite this experimental artefact, both the measured respiration rate and increase in the respiration was significantly greater in the sediment treatment than both the open and shaded control (p<0.001, Table 2).

Previous research has illustrated declines in photosynthesis and the P/R ratio during sediment exposure due to the combined effects of both turbidity and sedimentation [43]. Here, we were able to separate and quantify the effects, and found that low light levels caused a ∼13% decline in the P/R ratio, whereas sediment settling resulted in a further ∼7% reduction in the P/R ratio, largely due to increased respiration rates. Greater reductions in the P/R ratio have been observed during acute sediment stress events where coral photosynthesis and respiration rates declined by 43% to 64% and 13 to 23%, respectively [29]. These declines in respiration rates contrast with our chronic sediment exposure study and illustrate the significance of exposure time and sediment load on coral physiological responses. Sedimentary experimental conditions created here represent chronic sediment regimes on reefs in nearshore turbid reef environments and suggest that under these conditions reduced light accounts for 66% of the decline in P/R and sediment settling accounts of 33% of the decline. Future experiments that separate and quantify the effects of light and sedimentation on corals can be replicated for acute sediment stress events where corals are exposed to greater quantities of sediment for shorter time periods.

The influence of sediments on coral energetics varied between the two coral species, with *G. fascicularis* suffering from a greater decline in net photosynthesis than *G. somaliensis*. The decline in the photosynthesis in the sediment treatment was due to reductions in the maximum quantum yield. The maximum quantum yield is a measure of the photosynthetic efficiency of the photosystem II (PS II), hence a decline in the yield may limit the rate of photosynthesis at low light [25]. Note that the fluorescence yield and oxygen production can become decoupled, however, this typically occurs at high light levels (>300 PAR), whereas a positive linear relationship has been found at lower light intensities [60]. *G. fascicularis* had a lower maximum yield (0.51) than *G. somaliensis* (0.60) at the start of the experiment, so was less acclimated to low light conditions resulting in a greater decline in photosynthesis in the sediment treatment. Reductions in the maximum quantum yield due to sediment stress have also been measured in *Montipora* and *Porites*, where the decline in the yield was a function of sediment type and time of exposure [61]. Fine sediments were found to have the greatest influence, reducing the maximum yield by approximately 25% in 20 hours. Conversely, low light levels are known to increase the maximum quantum yield as corals photo-acclimate and increase their efficiency at utilising what light is available [25], [62], [63]. There was no significant change in the maximum yield of *G. somaliensis* in the shaded control although a decline in the yield was observed up until week 3 of the experiment, followed by an increase in week 4. The increase in yield in week 4 suggests that corals were photo-acclimating to the low light conditions by the end of the experiment. The maximum quantum yield of *G. fascicularis* in the shaded control also declined during the experiment, but by week 4 the yield had stabilised. If the experiment had continued we might have observed an increase in the yield (as with *G. somaliensis* in week 4) indicating that *G. fascicularis* was photo-acclimating to the low light conditions.

Greater respiration rates observed for *G. somaliensis* may, in part, be attributed to differences in coral morphology. Coral growth form, tissue angle, and polyp form all influence coral sediment tolerances [19], and some coral morphologies (e.g. those with tall polyps and convex colonies) substantially reduce the need for active removal thereby reducing energy expenditure [64]. A recent review of 77 published studies found that coral tolerances to turbidity and sedimentation were significantly related to growth form but not calyx size [30]. The morphology of *G. somaliensis* is characterised by many shallow calices (<5 mm) that form a relatively smooth surface [45]. Sediments are rapidly removed from its surface by active modification of the boundary layer through the projection of polyps above the colony surface and ciliary action [27]. Our results suggest that this combined sediment clearance method although effective in that it limits the amount of reduced light penetration by settled sediments, does cause a significant increase in respiration. In contrast, *G. fascicularis* has corallites (∼7 mm) that are cylindrical tubes linked via the coenosteum [45], and sediments are removed mainly through ciliary mechanisms [27]. Respiration rates for *G. fascicularis* in the sediment treatment were lower than for *G. somaliensis* and not significantly different from the shaded control, which suggests that less energy is required for ciliary action than polyp projection. Despite elevated respiration rates and associated sediment clearing, the yield of *G. somaliensis* was comparable to *G. fascicularis* by week 4 suggesting that both corals were experiencing photo-physiological stress.

In turbid reef environments spatial variations in rates of sediment resuspension and deposition are driven by local variations in hydrodynamics, with sheltered habitats typically dominated by sediment deposition and exposed habitats dominated by sediment resuspension [65]. These spatial variations in the sedimentary regime will drive coral species distributions depending on coral tolerances to sediments [16], [17]. Anthony and Connolly [43] attribute such species partitioning to increases in respiration rates with rising sediment load, which contrasts with an earlier study by Anthony and Fabricius [42] who stated that photosynthetic rates and heterotrophy were key drivers in habitat distribution of corals. Based on our results we postulate that coral species distributions along gradients of turbidity and sedimentation will depend on the interplay of individual tolerances to both sedimentation and light reduction. Coral tolerances to sediments are largely driven by their efficiency at sediment removal and how quickly they can photo-acclimate and/or switch to heterotrophy [32], [42]. Those corals that can remove sediments with minimum energy use (e.g. *G. fascicularis*) may be more suited to depositional environments whereas those coral species that can both rapidly remove sediments and photo-acclimate (e.g. *G.somaliensis*) may be more suited to reef habitats dominated by sediment resuspension. Such patterns of distributions of these two species are indeed confirmed by observations from inshore turbid reefs in Singapore [2] and on inshore reefs on the Great Barrier Reef [16], [36], [38], [66], emphasising the important balance between sediment deposition and resuspension in driving coral species distributions.

A central component of the present study was the silicon carbide mixture which provided an improved medium with which to test the direct (sedimentation) and indirect (reduced light) negative effects on corals without the additional confounding factors typically associated with natural sediments (e.g. nutrients, organics, and bacteria). However, the authors acknowledge that there may be some potential concerns when (a) substituting artificial for natural sediments, and (b) comparing the effects of reduced light from shading versus turbidity. Even though the silicon carbide powder used is inert, there may still be some scope for these sediments to act as vectors for microbes and, as such, future studies may consider it appropriate to pass sediments through ozone gas as a means of eliminating microbial contamination. There will also be differences in the surface integrity of the sediments and particle shapes between natural sediments and silicon carbide. However, given the fine particle sizes used in the study, comparable to that of silicon carbide powder used in previous sediment stress studies [19], particle shape and surface integrity are considered to be less important than ensuring that sediments are free of bacteria and nutrients, factors known to significantly influence coral physiology [31], [42]. A secondary experimental component is that light levels in the shaded control and sediment treatment were comparable. Light levels were controlled for, but there may have been some differences in the spectral composition of light, a key driver of photosynthesis [67], between the shaded and sediment treatments. The spectral composition of light has only relatively recently become a concern, and consequently has been assessed in a limited number of studies concerning corals and turbidity [68], [69], [70]. However, Telesnicki and Goldberg [70] found that there was no significant difference in the light spectral quality at low to high turbidity concentrations when using fine sediments composed of marl, a naturally occurring carbonate. These data would suggest that fine silicon carbide sediments, at low turbidity concentrations, would have a limited influence on the light quality and hence, light spectral quality would be comparable between the shaded control and sediment treatment.

Chronic sediment stress from increased sediment loading is a major threat to coral reef health, particularly for those reefs located close to shore. Managing coral reef health requires quantitative data on coral tolerances to sediments, both suspended and deposited, to generate predictive tools that can determine the impacts of increased sediment loading on coral reefs. This experiment successfully partitioned the effects of turbidity and sedimentation on coral energetics in chronic sedimentary conditions. All coral fragments survived the four week experiment, and no tissue mortality was observed, suggesting that these two coral species can tolerate the physical impacts of a chronic sedimentary regime of <27.4 mg cm^−2^ d^−1^ for at least one month. We found that under these sedimentary conditions, reduced light and sediment settling were responsible for 66% and 33% of the associated decline in the P/R ratio, respectively. In contrast, the response in yield to reduced light and sediments varied between species, most likely due to morphological differences, with either reduced light having a comparatively greater negative influence on yield and photo-physiological (*G. fascicularis*) or sediment settling alone resulting in significant physiological stress (*G.somaliensis*). Natural sediments, however, exert negative (e.g. bacteria) and positive (e.g. heterotrophy) pressures on corals, and direct comparisons between artificial and natural sediments would allow the additional assessment of the interplay between the physical and biological impacts of sediments on corals.
